# Antimicrobial Resistance in Agri-Food Chain and Companion Animals as a Re-emerging Menace in Post-COVID Epoch: Low-and Middle-Income Countries Perspective and Mitigation Strategies

**DOI:** 10.3389/fvets.2020.00620

**Published:** 2020-10-09

**Authors:** Samiran Bandyopadhyay, Indranil Samanta

**Affiliations:** ^1^ICAR-Indian Veterinary Research Institute, Eastern Regional Station, Kolkata, India; ^2^Department of Veterinary Microbiology, West Bengal University of Animal and Fishery Sciences, Kolkata, India

**Keywords:** backyard, COVID, food animals, mitigation, industrial food animal production, antimicrobial resistance

## Abstract

Antimicrobial resistance (AMR) leads to enormous financial losses from issues such as high morbidity, mortality, man-days lost, hospital length of stay, health-care, and social costs. In humans, over prescription of antimicrobials, which is presumably higher during COVID, has been identified as the major source of selection for antimicrobial resistant bacteria; however, use of antimicrobials in food and companion animals, fish, and vegetables, and the environmental resistance gene pool, also play important roles. The possibilities of unnecessary use of antibiotics as prophylaxis during and after COVID in livestock and companion animals exist in low-and middle-income countries. A considerable loss in gross domestic product (GDP) is also projected in low-and middle-income countries (LMICs) due to AMR by the year 2050, which is further going to be reduced due to economic slowdown in the post-COVID period. Veterinary hospitals dedicated to pets have cropped up, especially in urban areas of LMICs where use of antimicrobials has also been increased substantially. The inevitable preventive habit built up during COVID with the frequent use of hand sanitizer might trigger AMR due to the presence of cross-resistance with disinfectants. In LMICs, due to the rising demand for animal protein, industrial food animal production (IFAP) is slowly replacing the small-scale backyard farming system. The lack of stringent regulations and monitoring increased the non-therapeutic use of antimicrobials in industrial farms where the persistence of antimicrobial resistant bacteria has been associated with several factors other than antimicrobial use, such as co-resistance, cross-resistance, bacterial fitness, mixing of new and old animals, and vectors or reservoirs of bacterial infection. The present review describes types of antimicrobials used in agri-food chains and companion animals in LMICs with identification of the gap in data, updated categories of prevalent antimicrobial resistant bacteria, the role of animal farms as reservoirs of resistant bacteria, and mitigation strategies, with a special focus on the pivotal strategy needed in the post-COVID period.

## Introduction

Human and animal populations are at risk of cross-transmission of zoonotic bacteria via direct contact due to close proximity with food animals, companion animals, live wildlife markets, environmental contamination, and the intake of contaminated animal origin food items. The situation becomes more complicated due to cross-transmission of antimicrobial resistance (AMR) determinants along with the infection. In humans, over prescription of antimicrobials is the major source of selection for antimicrobial resistant bacteria, but use of antimicrobials in food animals and, moreover, the environmental resistance gene pool (“resistome”) also play important roles in this complex multi-factorial state of affairs. Recently, the bacteriostatic antimicrobial (azithromycin) was recommended in synergism with hydroxychloroquine against SARS-CoV2 in treatment protocols in several countries despite the dearth of precise clinical evidence (1, 2). The recent systematic review revealed use of antibiotics in 70% of COVID patients, mostly in Asian countries, although only 10% of them had a bacterial co-infection (3). Even the World Health Organization (WHO) warned against the overuse of antibiotics during the pandemic with the statement: “The COVID19 pandemic has led to an increased use of antibiotics, which ultimately will lead to higher bacterial resistance rates that will impact the burden of disease and deaths during the pandemic and beyond” (4). The Post-COVID epoch may add complexities to the AMR perspective, as antibiotics might be considered as a prophylactic measure among the community, especially in LMICs where antibiotics are easily available at the counter without prescriptions (5). The manufacturers of azithromycin are already facing difficulties to meet the ever-increasing demands (6). Prophylactic antimicrobial therapy in food and companion animals may witness a steep rise during and after the COVID episode, particularly in LMICs, even if it is not recommended in many countries (7). The situation becomes catastrophic as the companion animal practitioners prefer human antibiotics for their better quality and easy availability.

AMR leads to enormous financial losses associated with high morbidity, mortality, man-days lost, hospital length of stay (LOS), direct health-care costs, and the social costs of infection (8). About 700,000 deaths per year were attributed to AMR alone, which is more than the toll caused by malaria, acquired immunodeficiency syndrome (AIDS), and tuberculosis (9). The World Health Organization (WHO) identified eight pathogens relevant to AMR, including five bacteria (*Klebsiella pneumoniae, Escherichia coli, Staphylococcus aureus, Neisseria gonorrhoeae*, and *Mycobacterium tuberculosis*) (10). Among them, third-generation cephalosporin-resistant and carbapenem-resistant *Enterobacteriaceae* (CRE, e.g., *Escherichia coli* and *Klebsiella pneumoniae*) alone were reported to cause 6.4 million bloodstream infections and 50.1 million serious infections worldwide in a year (11). A recent estimate suggested 33,000 annual deaths due to AMR in the European Union and European Economic Area (12). Additional treatment costs and losses due to methicillin-resistant *Staphylococcus aureus* (MRSA) and third-generation cephalosporin-resistant and ESBL-producing *Enterobacteriaceae* ranged between 1,732 and 9,726 USD and 2.54–6.8 days per case, respectively (8). For the United States alone, average national health care expenditure was estimated at around 2.2 billion USD due to AMR (13).

Addressing AMR in developing countries was considered crucial by the United Nations to achieve sustainable development goals (SDGs) associated with poverty and hunger alleviation and the improvement of health and economic growth (14). In LMICs, the current rate of AMR-related infections is high and is projected to grow more rapidly than in developed countries. A substantial portion (40–60%) of human bacterial infections in Brazil, the Russian Federation, and India is associated with resistant bugs in comparison to developed countries (17%) (15). In LMICs, the direct and prominent effects of AMR include increased mortality, in addition to higher morbidity and economic losses (16). The recent projection about the financial vulnerability of LMICs revealed that an additional 19 million people are going to fall into great poverty by 2030 due to AMR producing direct impacts on labor productivity (neat GDP produced by 1 h of labor) and increased health care costs (17). A considerable loss in GDP is also projected in low-income countries due to AMR by the year 2050, which is further going to be reduced due to economic slowdown in a post-COVID scenario (18).

The present review describes types of antimicrobials used in food animals, companion animals, aquaculture, and vegetables in LMICs, categories of prevalent antimicrobial-resistant bacteria in LMICs, the role of industrial and backyard farms as a reservoir of resistant bacteria in LMICs, and mitigation strategies with special reference to a post-COVID scenario.

## Definitions and Uses of Antimicrobials

Antimicrobials (AM) are substances of natural, semisynthetic, or synthetic origin that kill or inhibit the growth of a microorganism but cause little or no damage to the host cells. Antibiotics (AB) are low molecular weight antimicrobials produced by a microorganism that at low concentrations inhibit or cause lysis of other microorganisms. WHO made a list of medically important antimicrobials (MIA) and classified them into three categories, critically important antimicrobials (CIA, highest priority CIAs and high priority CIAs), highly important antimicrobials (HIA), and important antimicrobials (IA), based on five criteria (19). Similarly, the World Organization for Animal Health (OIE) determined the degree of importance for classes of veterinary antimicrobial agents based on antimicrobial class, use in treatment of serious animal diseases, and availability of alternative antimicrobial agents (20). Different classes of important veterinary antimicrobials, mechanism of action, indication, and mechanism of resistance are described in Tables 1, 2.

**Table 1:** Characteristics of selected veterinary important antimicrobials.

**Antimicrobials**	**Mechanism of action**	**Indications**		**WHO classification**	**OIE classification**	**Resistance mechanism**
		**Human**	**Animals**			
Sulfonamides and Potentiated Sulfonamides Sulfachloropyridazine (sui & bov) Sulfadiazine (can and fel) Sulfadimethoxine (bov, can and fel) Sulfamethazine (bov, sui, can and fel) Sulfamethoxazole (can and fel) Sulfaquinoxaline (Calves, small ruminants and poultry) Ormetoprim + sulfadimethoxine (can and fel) Trimethorprim + sulfadiazine (equ, can and fel) Trimethoprim + sulfamethoxazole (equ, can and fel) Trimethoprim + sulfadoxine (bov)	Sulfonamide mimics paraamino benzoic acid (PABA) as a false substrate and trimethoprim/ormetoprim inhibits dihydrofolate reductase enzyme. Altogether, these compounds inhibit the synthesis of dihydrofolic acid, an important co-enzyme for many complex biochemical pathways in bacteria, including DNA synthesis.	sulfamethoxazole–trimethoprim combination (co-trimoxazole) indicated in UTI infections, prostatitis, chronic bronchitis and invasive salmonellosis	Bacterial (*Staphylococcus* spp., Corynebacterium, Nocardia asteroides, Stenotrophomonas maltophilia, and bacteria of the Enterobacteriaceae (Klebsiella, Proteus, Enterobacter, and *Escherichia coli*), Pasteurella) and protozoal (Histophilus, Toxoplasma, and coccidia.) infections Pneumonia, intestinal infection (coccidian), soft tissue infection, UTI Sulfaquinoxaline is indicated in coccidial enteritis.	HIA	VCIA	Efflux pumps and changes in the target enzymes
Penicillins Natural penicillins Penicillin G – (bov, sui,ovi and equ) Aminopenicillins Ampicillin (can, fel, equ) - AMC Ampicillin + sulbactam (can, fel, equ and ruminants) –A/S amoxicillin (bov, equ, can, fel) amoxicillin + clavulanate potassium (can and fel) Antistaphylococcal penicillins (e.g., oxacillin, oxacillin, cloxacillin, and dicloxacillin) Limited clinical use COX: Mastitis D/C: Can and fel Extended-spectrum penicillins (e.g. piperacillin) TI/TIC (can, fel and equ) CB (Can and fel) PI (Can and fel)	Penicillin and cephalosporins – β-lactam drugs inhibit bacterial cell wall synthesis by interfering the transpeptidation reaction.	Penicillins: Active against nonpenicillinase-producing *Staphylococcus, Streptococcus*, few Gram-negative bacteria - Arcanobacterium, Mannheimia haemolytica, Listeria monocytogenes, and Pasteurella, anaerobes *Fusobacterium, Peptococcus, Peptostreptococcus*, some strains of Bacteroides and Clostridium, *Bacillus anthracis* spirochetes (Leptospira, and Borrelia burgdorferi). Aminopenicillins are active against the bacteria resistant to Penicillin G – *Enterobacteriaceae, Pseudomonas, Bacteroides fragilis*, and penicillinase producing *Staphylococcus* spp., Antistaphylococcal penicillins penicillin G and the aminopenicillins resistant penicillinase producing *Staphylococcus* spp. Few other gram-positive and gram-negative bacteria and spirochetes. Extended-spectrum penicillins Gram-negative aerobic and anaerobic Bacteria; many strains of Enterobacteriaceae and Pseudomonas	Penicillin G – (Anthrax, BQ, HS in large animals) Aminopenicillins AMP: (UTI, pneumonia, wound) A/S: (acute infection – pneumonia, sepsis and infections caused by ESBL pathogens, prophylaxis in neutropaenic patients) AMX: (UTI, soft tissue infection, pneumonia) AMC: (skin, UTI, respiratory and wound infection) Antistaphylococcal penicillins COX: used in intramammary preparation for treating mastitis D/C: β-lactamase producing Staphylococcus Extended-spectrum penicillins TI/TCC, CB, PI, PIT Soft tissue/ bone infection, pneumonia (synergistic with aminoglycosides) Ampicillin-resistant bacteria, Pseudomonas aeruginosa, Clavulanate potentiates its action against Gram-negative bacteria and *Staphylococcus*	HIA	VCIA	Mediated by production of enzymes like β-lactamases that render the penicillins/cephalosporins by hydrolysis of β-lactam rings. *Staphylococcus* can become resistant by mutating the penicillin binding proteins (PBP2a) which have a reduced affinity to β-lactam drugs.
Cephalosporins First generation Cefacetrile (bov) Cefalexin (bov,cap, equ, ovi, sui, can, fel) Cefalothin (can, fel, equ) Cephapirin (bov) Cefazolin (bov, cap, ovi, can, can, fel) Cefalonium (bov, cap, ovi) Cefadroxil (can, fel, equ) Second generation Cefuroxime (bov) Cefaclor (can, fel) Cefoxitin (can, fel, bov and equ)		Active against most of the gram positive bacteria except *Enterococcus*. Greater activity against *Enterobacteriaceae* than penicillin. 2nd generation cephalosporins are more active against *Enterobacteriaceae* Cephamycin (cefoxitin) groups are also effective in anaerobic infection.	Skin, soft tissue, respiratory tract and urinary tract infections, wound, abscess Eft is useful in porcine respiratory disease complex (PRDC), bovine respiratory disease complex (BRDC) and bovine mastitis.	HIA	VCIA	Narrow-spectrum β-lactamases can neutralize early generation cephalosporins but not the higher generation cephalosporins. Extended-spectrum β-lactamases produced by some strains of Gram-negative bacteria can deactivate 3rd and 4th generation cephalosporins.
Third generation Cefoperazone (bov, cap, ovi) Ceftiofur (avi, bov cap, equ, ovi, lep, can, fel) Ceftriaxone (avi, bov, ovi, sui) Cefpodoxime (can, fel and equ) Cefotaxime (can, fel and equ) Fourth generation Cefquinome (bov, cap, equ, ovi, sui) Potentiated cephalosporins (CAZ/CTX with clavulinic acid, sulbactum or tazobactum)		3rd generation cephalosporins are more active against Gram-negative bacteria than the 1st and 2nd generations which are rendered ineffective by production of β-lactamase. CPZ and CAZ are useful in infections caused by *Pseudomonas* Eft is active against Pasteurella multocida, Mannheimia haemolytica, *Histophilus somnus, Fusobacterium* necrophorum, *Actinobacillus, Salmonella cholerasuis, Streptococcus* suis		CIA		
Tetracyclines Chlortetracycline (avi, bov, cap, equ, lep, ovi, sui, can, fel) Oxytetracycline (api, avi, bov, cam, cap, equ, lep, ovi, pis, sui, can, fel) Tetracycline (Api, avi, bov, cam, cap, equ, lep, ovi, pis, sui, can, fel) Doxycycline (avi, bov, cam, cap, equ, lep, ovi, pis, sui, can, fel) Minocylcine (can, fel)	Tetracycline binds to the 30S ribosomal subunit and interferes with the interaction of aminoacyl-tRNA with mRNA leading to bacterial protein synthesis inhibition.	Possibly, tetracycline has the broadest spectrum of activity being effective against mycoplasma, Rickettsia, chlamydia and blood protozoa apart from bacteria. Tetracyclines are indispensible drugs for treating Ehrlichiosis, anaplsmosis and as an adjunct therapy in theileriosis – both are endemic in many Asian and African countries. Use to treat infections caused by Pasteurella multocida, Mannheimia haemolytica, *Histophilus somnii*	Pneumonia including BRDC, PRDC, enteritis, abscess, skin and soft tissue infection. In pigs these drugs are useful in atropic rhinitis, *Mycoplasma* infection and pneumonic pasteurellosis. In foals tetracyclines are used to treat angular deformities, possibly for their anti-inflammatory, chondroprotective, and antiarthritic effects.	HIA	VCIA	Resistance is mediated by energy dependent efflux of the drugs and alteration of binding sites of tetracycline at the 30S ribosomal units.
Aminoglycosides Streptomycin (api, avi, bov, cap, equ, lep, ovi, pis, sui) Dihydrostreptomycin (avi, bov, cap, equ, lep, ovi, sui) Gentamicin (avi, bov, cam, cap, equ, lep, ovi, sui, can, fel) Amikacin (equ, bov, can and fel) Neomycin (api, avi, bov, cap, equ, lep, ovi, sui, can, fel) Kanamycin (api, avi, bov, cap, equ, lep, ovi, pis, sui, can, fel) Paromomycin (cap, ovi, lep) Apramycin (avi, bov, lep, ovi, sui)	Its irreversible attachment to 30S ribosomal subunit leads to interruption in mRNA translation process. This ultimately leads to premature termination or faulty protein synthesis due to misreading of genetic codes.	Effective against Gram-negative bacteria- *Enterobacteriaceae* and *Pseudomonas aeruginosa*. Efficacy against Gram-positive bacteria like *Staphylococcus* is limited. Anaerobic pathogens are inherently resistant.	Useful in septicaemias; digestive, respiratory and urinary tract infections. Few drugs have specific indication like apramycin in swine colibacillosis (pig scours). Paromomycin is useful in protozoal gastrointestinal infections.	CIA	VCIA	Anaerobes are inherently resistant as the drugs require oxygen for entry into the cell. Resistance mechanism involve alteration in the cell surface receptor to slow down or block the passage of the drugs, changes at the drug attachment sites (30S ribosome) and enzymatic degradation. Amikacin being unaffected by many of the hydrolyzing enzymes is more effective than other aminoglycosides in controlling infections caused by resistant bacteria.
Phenicol Florfenicol (avi, bov, cap, equ, lep, ovi, pis, sui, can, fel) Thiamphenicol (avi, bov, cap, ovi, pis, sui, can, fel)	Phenicols are bacteriostatic agents – phenicols interfere the peptidyltransferase enzyme activity at 50S ribosoma subunit leading to protein synthesis.	Effective against *Mannheimia haemolytica, Pasteurella multocida, Histophilus somni, Fusobacterium necrophorum, Bacteroides, Actinobacillus, Salmonella cholerasuis and Streptococcus suis, Aeromonas salmonicida*	Respiratory infections in poultry, BRDC, SRDC, foot rot, acute interdigital necrobacillosis and infectious pododermatitis	HIA	VCIA	Resistance mediated by a variety of mechanism *viz.*, efflux pumps, enzymatic modifications by rRNA methyltransferases, and chloramphenicol acetate esterases and inhibition of intracellular drug transport
Macrolides Azalide Azithromycin*(equ, sui, can, fel) Tulathromycin (bov, cap, lep, ovi, sui) Macrolides C14 Erythromycin (api, avi, bov, cap, equ, lep, ovi, pis, sui, can, fel) Macrolides C16 Spiramycin (api, bov, cap, equ, lep, ovi, pis, sui, can, fel) Tilmicosin (avi, bov, cap, lep, ovi, sui) Tylosin (api, avi, bov, cap, lep, ovi, sui, can, fel)	By binding to the 50S ribosomal subunit at 23sRNA site, macrolides inhibit the protein systhesis. Tilmicosin is effective in BRDC by reduced expression of PGE_2_ and release of anti-inflammatory cytokines.	Gram-positive infections mainly, Mycoplasma, Rhodococcus, Chlamydophila Mycoplasma, Arcanobacterium, Erysipelothrix, Bordetella, and Bartonella Moraxella, Serpulina Lawsonia	Respiratory infection, hemorrhagic digestive diseases- swine dysentery and proliferative enteropathy (sui), liver abscess, pododermatitis (bov) Additionally, tylosin is effective in pink eye	CIA	VCIA	Resistance is mediated by *mef* gene governed drug efflux system, drug inactivating enzymes and modification of the drug binding sites at 50S ribosome (erm genes)
Lincosamides Clindamycin (can, fel) Lincomycin (api, avi, bov, cap, ovi, pis, sui, can, fel) Pirlimycin (bov)	Inhibit protein sysnthesis by binding with 50S ribosomal subunit.	Staphylococcus, Streptococcus, Actinomyces, Nocardia, Mycoplasma and Cornybacterium, Erysepelothrix, Leptospira, *Bacteroides fragilis, Fusobacterium* spp., *Peptostreptococcus* spp., and *Clostridium perfringens* Babesia Toxoplasma.	Gram-positive or anaerobic infections in oral cavity, skin, soft tissue, respiratory tract, protozoal infection Lincomycin is used in pyoderma in pets and mycoplasma infections in pigs and poultry and infectious arthritis and hemorrhagic enteritis in pigs			MRSP from dogs are usually resistant while community acquired MRSA are susceptible. Resistance driven 23sRNA methylation encoded by erm gene is the most common mechanism apart from drug efflux pumps (mef) and enzymatic modification of the drugs.
Quinolones Quinolone 1G Fluoroquinolones Danofloxacin (avi, bov, cap, lep, ovi, sui,) Difloxacin (avi, bov, lep, sui, equ, dog) Enrofloxacin (avi, bov, equ, lep, ovi, pis, sui, can, fel) Orbifloxacin (bov, sui, can, fel) Pradofloxacin (fel) Marbofloxacin (avi, bov, equ, lep, sui)	Quninoloes are bactericidal by inhibition of DNA replication and transcription. DNA gyrase encoded by *gyrA* and topoisomerase IV encoded by *ParC* and *ParD* are targets of this group of drugs.	Fluoroquinolones are broad-spectrum drugs; however, they are more active against the Gram-negative bacteria like *Enterobacteriaceae*. Gram-positive bacteria- *Staphylococcus* are variably susceptible. Marbofloxacin and pradifloxacin are more effective against Gram-positive bacteria. *Pseudomonas* is not invariably susceptible. Besides, this group is effective against *Pasteurella multocida, Mannheimia haemolytica, Histophilus somni* and other intracellular organisms - *Rickettsia* spp., *Chlamydia*, and *Mycobacterium* spp. and *Mycoplasma* spp	BRDC, septicemia, UTI, gastroenteritis, Enrofloxacin is effective against many Rickettsia but not against *Ehrlichia* CRD in poultry	CIA	VCIA	Decreased permeability, efflux pumps, altered targets, plasmid-mediated resistance were recorded. Mutation in the quinolone resistance determining region – gyrA, ParC and ParE is responsible for decreased affinity of quinolones or fluoroquinolones to gyrase and topoisomerase.
Peptides Bacitracin (avi, bov, lep, sui) Colistin	Bacitracin kills the bacteria by interfering with cell membrane function, suppressing cell wall formation and inhibiting protein synthesis in the presence of divalent cations, such as zinc. Colistin or polymixin interacts with LPS of Gram-negative bacteria leaving a porous cell-membrane and eventually cell-death.	Bacitracin is mainly effective against Gram-positive bacteria Colistin is useful in digestive diseases by Gram-negative infections	Bacitracin is useful in necrotic enteritis in poultry. With the concerns over selection of drug-resistant bacteria, use of colistin and Bacitracin Methylene Disalicylate (BMD) is under scrutiny and has been banned by various countries including India.	Colistin (CIA)	VHIA	Colistin-resistance is mediated by changes in the overall charges LPS of the bacterial cell membrane brought about by plasmid mediated gene *mcr* or alternation in two component signaling system. Resistance to bacitracin is rare.
Ionophores Lasalosid (avi, bov, lep, ovi) Maduramycin (avi) Monensin (avi, api, bov, cap) Salinomycin (avi, lep) Naracin (avi) Semiduramicin (avi)	Ionophores cause ion imbalance in bacterial cell making them energy deficient		Mainly used in treatment of coccidiosis In ruminants, it decreases methane production and better utilization of carbohydrate and protein utilization. Ionophores are also useful in liver abscess and rumen acidosis or bloat as they prevents propionic acid production.		VHIA	Bacteria may become temporarily ionophores-resistant by shedding out of cell membrane or by forming a glycoprotein armor (glycocalyx) around their body (Russell and Houlihan 2003).
Novobiocin	By binding to DNA gyrase, it blocks adenosine triphosphatase (ATPase) activity.	*S. aureus* and CoNS including MRSA	Mastitis and sepsis in fish		VIA	Mediated by a mutation in the target – gyrB (21)
Avilamycin	Avinamycin binds to the 50S ribosomal unit to prevent bacterial protein synthesis	Gram-positive bacteria like Clostridium perfringens	Necrotic enteritis in poultry, and enteric disease in pig and rabbits.		VIA	
Peuromutilin Tiamulin (Avi, Cao, Lep, Ovi, Sui)	It is a bacteriostatic antibiotic and inhibits the protein synthesis by binding to the 50S ribosomal subunit	Effective against gram-positive bacteria, mycoplasmas, and anaerobes, including Brachyspira hyodysenteriae.	It is also clinically effective in treatment of swine dysentery and mycoplasmal arthritis, respiratory diseases of pigs and poultry		VHIA	Chromosomal (mutations in the 23S rRNA and *rplC* genes) and plasmid-mediated (vga and cfr genes) resistance were reported (22)

*AVI, avian; EQU, Equine; API, bee; LEP,Rabbit; BOV, bovine; OVI, Ovine; CAP, caprine; PIS, Fish; CAM, camel; SUI, Swine; Can, Canine; Fel, Feline; VCIA, Veterinary Critically Important Antimicrobial Agents; VHIA, Veterinary Highly Important Antimicrobial Agents; VIA, Veterinary Important Antimicrobial Agents; CIA, Critically Important Antimicrobials; HIA, Highly Important Antimicrobials*.

**Table 2:** Characteristics of selected veterinary important antifungals.

**Antifungal**	**Mechanism of action**	**Indications**	**Resistance mechanism**
**Griseofulvin**	Interaction of griseofulvin with mitotic spindles leads to cell cycle arrest and finally cell death.	Treatment of dermatophytosis Effective against *Microsporum* spp., *Trichophyton* spp., and *Epidermophyton*	Many are not responsive to griseofulvin due to the intrinsic resistance owing to the absence of energy dependent uptake of the drug is present in many fungus
Azole compounds Imidazole Clotrimazole (can, fel), Miconazole (can, fel) Ketoconazole (can, fel) Triazoles Fluconazole (avi,equ, can, fel) Voriconazole (avi, equ, can, fel)	Impairs the ergosterol synthesis by inhibition of lansosterol C14 demethylase enzyme (CYP51A/Erg11p)	Ketoconazole: Effective against *Candida, Malassezia pachydermatis, C. immitis, H. capsulatum*, and *B. dermatitidis* and most dermatophytes Useful in canine blastomycosis, histoplasmosis, cryptococcosis, and coccidioidomycosis Itraconazole and fluconazole are more effective than ketoconazole. Proven efficacy of itraconazole against Aspergillosis and in *Malassezia* dermatitis gives it an edge over ketoconazole. Voriconazole is also effective against Aspergillus and Fusarium Generally, miconazole used as topical agent (cream or spray) in canine dermatophytosis. Systemic use of clotrimazole is limited because of poor oral absorption. However, topical administration is effective in otitis externa caused by *Malassezia pachydermatitis*. Clotrimazole is effective in nasal aspergillosis and caniduria in small animals.	Increased biosynthesis of lanosterol C14α-demethylase, mutation at the target site (ERG11), efflux pump mediated drug expulsion and alternate pathways to replace ergosterol with other compounds are the major azole-resistance mechanisms.
Terbinafine (avi, can, fel)	Inhibits ergosterol biosynthesis by interacting squaline epoxidase enzyme	Dermatophytosis, topical forms are useful	Terbinafine resistance is uncommon; however, mutation of squalene epoxidase was recorded to mediate such resistance in clinical isolates of dermatophytes.
Polyene compounds Nystatin Natamycin Amphoterecin B	Binds with ergosterol of the fungal plasma membrane causing leakage of essential nutrients and cell death.	Nystatin as topical agent, oral and intestinal candidiasis Natamycin is useful in keratomycosis, nasal aspergillosis, guttural pouch mycosis and dermatophytosis in horses. Amphoterecin B: Histoplasma capsulatum, *Cryptococcus neoformans, Coccidioides immitis, Blastomyces dermatitidis, Candida* spp., and various species of Aspergillus.	Mutation in *ERG3* gene which is responsible for ergosterol biosynthesis leads to incorporation of other sterols in plasma membrance and polyene fails to act on them.

## Use of Antimicrobials in the Agri-Food Chain and Companion Animals

### Vegetable Production

Some antibiotics are used to protect profitable fruits, vegetables, ornamental plants, and crops from bacterial diseases. The manure used in green houses and soils acts as an additional source of antimicrobial residue for fruits and vegetables which should be treated before direct use (manure composting or aerobic treatment). Although the studies could not detect any AMR-associated bacteria in vegetables as such (23), residues of tetracycline, virginiamycin, tylosin, monensin, and sulfamethazine could be detected in vegetables (24) and in green-house soil following manure application (25). The untreated irrigation water used for the production of vegetables, fruits, and crops was identified to contain AMR determinants (*tet, pAmpC*) in South Africa, which is an indirect indication, although the precise AMU data for vegetable production is not available in LMICs (26).

### Food Animals

In food animals, antimicrobials are used for several purposes such as therapy, prophylaxis, metaphylaxis, and promotion of growth. Therapeutic usage of antimicrobials is difficult to discontinue, as it not only saves the animal's life but also decreases the zoonotic pathogen load in the environment and reduces methane production by livestock (monensin) (27). Nevertheless, the use of CIAs should be optimized and should only be allowed only in emergency or special infectious conditions, like higher generation or potentiated cephalosporins (cefoperazone, ceftiofur, ceftriaxone, cefquinome) in the treatment of septicemias, respiratory infections, and mastitis; aminoglycosides in septicaemias, severe digestive, respiratory, and urinary tract infections and in *Pseudomonas aeruginosa* infections; fluoroquinolones in the treatment of septicaemias, respiratory, and enteric diseases; and macrolides in *Mycoplasma* infections in pigs and poultry, *Lawsonia intracellularis* in pigs, and hepatic abscess in cattle due to *Fusobacterium necrophorum* [Table 1, (28)].

Use of colistin, the last resort antibiotic in human medicine, in poultry and pigs in China recently caused the emergence of colistin-resistant bacteria possessing the novel resistance gene *mcr-1* (29). Furthermore, other variants of *mcr* were found in animals and humans, viz., *mcr-2, mcr-3, mcr-4*, and *mcr-5* (30). China and India recently banned the use of colistin as a growth promoter in food animals (31, 32). Prophylaxis or prevention is the administration of antimicrobials to healthy animals considered to be at risk to prevent the future occurrence of infection, while the prophylactic use in healthy food animals, including poultry, is not yet scientifically validated (prohibited in Europe), except in blanket therapy. Prophylactic antibiotic use should not be a substitute for improper biosecurity and inadequate husbandry conditions of the farms. Metaphylaxis refers to the use of antimicrobials in the infected and healthy but at risk animals of the same herd to prevent the spread of the infection. It is preferred in large herds where separation of healthy animals from infected ones is difficult based on clinical signs and rectal temperature (33).

The growth promotion effect of antimicrobials in animals is doubtful as it is observed that satisfactory effects can be produced only during the early stages of animal production or in sub-optimal hygiene conditions (34). Many non-MIA, such as bacitracin, bambermycins, and carbadox, are currently used for growth promotion. Although following the immediate ban of antibiotics as growth promoters in Europe, a few clinical conditions, such as post-weaning scours, occurred in pigs with higher frequency (35). The meta-analysis showed that the restriction of antibiotics as growth promoters in animals reduced the occurrence of antimicrobial-resistant bacteria in animals and humans having close contact with the animals, but the analysis could not reveal the effect on the community (36). Other studies could not establish any strong evidence that the restriction of antibiotic use in animals reduced the occurrence of antimicrobial-resistant bacteria in the human population (37).

Use of MIA in animals for growth promotion, prophylaxis, and even metaphylaxis is considered as inappropriate antimicrobial usage (IAMU) by international agencies such as the World Health Organization (WHO), Food and Agricultural Organization (FAO), and World Organization for Animal Health (OIE). Recently, WHO recommended complete cessation of MIA use in healthy animals for prophylaxis or growth promotion (19). Among the MIA sold for animal production, tetracycline and penicillin constitute 32% and 6% of total sale on weight basis in the United States, 29% and 25% in European Union, 51% and 8% in Canada, 47% and 12% in Japan, and 9% and 9.8% in Australia, respectively. Cephalosporins are the MIA used with the lowest share (> 1%) among the sold antibiotics for animal production in the studied countries (38). Non-therapeutic antimicrobial use is common among food animals, like prophylactic intramammary antimicrobial infusion in the form of penicillin or β-lactam in dairy animals, macrolides in beef feedlot cattle for respiratory illness, and tylosin in beef calves to prevent liver abscesses. Likewise, tylosin and tetracyclines are common antibiotics used as feed additives in 88% of the growing pigs in the USA (39). In the USA, about 74% of farm-animals that received antibiotics were in feed and 21% in the drinking water, and the sale of medically important antibiotics was three-time higher in the animal sector than in human beings (40). There is a considerable deficit of data on AMU in food animals, including poultry, from LMICs due to a lack of national-level surveys (41). The systematic study revealed the maximum use of tetracyclines followed by aminoglycosides, beta-lactams, macrolides, arsenicals, fluoroquinolones, ionophores, penicillins, polymyxins, polypeptides, and sulfonamides, but species-level consumption data from LMICs are largely unavailable (42). China exponentially increased the use of antimicrobials for animal production from 6 million kg in 2001 to 84.2 million kg in 2013, which is substantially higher than the United States and Europe (43). Tetracycline and penicillin constitute 33 and 20%, respectively, of total MIA sale for animal production in the Republic of Korea (38). Southeast Asia (SEA) is a group of rapidly developing LMIC (except Singapore, Brunei, and Laos) that shares a linked economy through export of aquaculture (Vietnam, Thailand, and Indonesia) and poultry (Thailand) products (44). The meta-analysis of the literature published from SEA (mostly Vietnam and Thailand) identified the use of amoxicillin in most of the farms, followed by enrofloxacin, norfloxacin, doxycycline, ampicillin, colistin, neomycin, gentamicin, tylosin, trimethoprim, florfenicol, erythromycin, chloramphenicol (although banned in Vietnam), sulfamethoxazole, and chlortetracycline for the production of pigs, chicken, and fish (45). The quantitative analysis revealed therapeutic use of 46 mg of antimicrobial compounds (penicillin, lincosamide, quinolone, and sulphonamides with trimethoprim) per kg of live pig and 52–276 mg per kg of live chicken in pig and poultry farms in Vietnam (23). For growth promotion, 286.6 mg and 77.4 mg of antimicrobials were used with feed to produce 1 kg of pork and chicken, respectively. The feeds of chickens and pigs in SEA mostly contained non-MIA groups of antibiotics, such as bacitracin (15–24% of feed formulations), enramycin (enduracidin), and florfenicol, except a single study from Vietnam which identified critically important antibiotics (colistin, amoxicillin, and neomycin) in chicken feed (25). Many of the antibiotics that are being used in food-animals for non-therapeutic purpose are not clinically relevant in human medicine but they may still confer cross or co-resistance to MIAs. Further, many of the antibiotics, like colistin (polymyxin), ardacin, avoparcin (glycopeptides), and virginiamycin (streptogramins), were classified as highest priority antimicrobials (polymyxin and glycopeptides) or highly important antimicrobial (streptogramins) (46). The global average annual consumption of antimicrobials per kilogram of animal produced was 45 mg/kg, 148 mg/kg, and 172 mg/kg for cattle, chicken, and pigs, respectively (24). The World Organization for Animal Health (OIE) estimated the amount of antimicrobial agents used in animals and detected an increase from 98.97 mg/kg in 2014 to 144.39 mg/kg in 2016 (47). Further increases in animal protein demand during and post the COVID period to boost immunity will increase meat production with higher intensity. Country-specific AMU surveillance data will allow for the scenario to be realistically predicted.

### Companion Animals

Companion animals are not directly linked to the human food chain and possibly manage to escape the hunt for AMR drivers across the globe, although isolated studies on AMR bacteria surveillance have indicated the tip of the iceberg (48). With growing concern in modern society over pet welfare, more and more affluent families started treating pets almost like family members. Veterinary hospitals dedicated to pets have cropped up, especially in urban areas of LMICs. Keeping pace with the increasing pet-health care facilities, the use of antimicrobials has also increased substantially and multi-drug resistant bugs, like MRSA, methicillin-resistant *Staphylococcus intermedius* (49), carbapenem-resistant *Enterobacteriaceae* (CRE), and even colistin-resistance *E. coli*, are also being detected in China (50). The last group reported a steady increase in recovery of MDR and β-lactam- and fluroquinolone- resistant *E. coli* from pet animals in China over 5 years (2012–17), possibly due to increased β-lactam usage in pet clinics. The situation became catastrophic during the COVID period due to non-availability of qualified companion animal practitioners (associated with prolonged lockdown in few countries), over-prescription in telehealth consultations without laboratory-based diagnosis, easy accessibility of medicines from online pharmacies, and the preference for human antibiotics for their better quality and easy availability, especially during the break-down of the pharmaceutical supply chain. More elaborative studies are needed to address the issues in LMICs.

### Aquaculture

**Figure 1:**
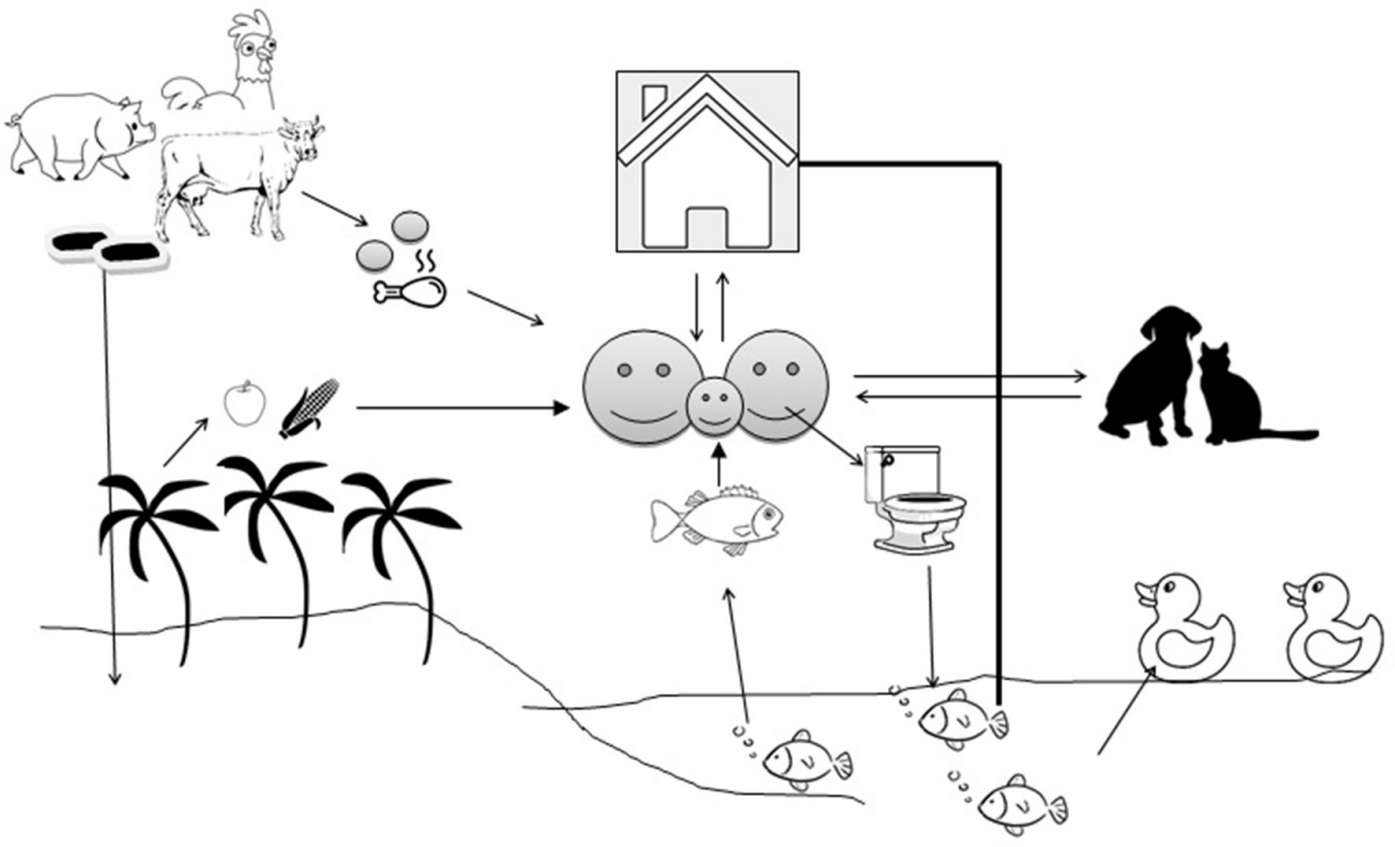
Transmission of antimicrobial resistance genes between the environment and animal/human compartments. The arrows indicate the flow of antimicrobial resistance genes.

In LMICs, open water aquaculture systems, such as ponds, are subjected to heavy pollution due to domestic and household activities, wastewater discharge, animal activities, and livestock manure fertilization. The pollution can significantly impact the microbial community and diversity of the pond water (51). Aquatic bodies are at the receiving end where untreated human and animal wastage manure are dropped and the aquatic bodies become ideal hosts for bacterial flora (52). Antimicrobial resistance genes (ARGs) are being exchanged across the bacterial species irrespective of their source and host specificity (53) (Figure 1). Antibiotics are applied *en masse* (metaphylactic) in fish as individual treatment is impractical, which also exposes the non-targets to such treatment (54). Fish generally excrete out most of the antibiotics into the water and sediment due to poor gut absorption and the waterbodies become storehouses for antibiotics, which exert selection pressure on the microflora (55). In the aquatic environment, transduction facilitates the lateral transfer of ARGs like β-lactamase (56) and humans can pick up resistances faster from aquatic sources than from terrestrial animals. Prophylactic use of antimicrobials in intensive/semi-intensive aquaculture is on the radar, since prolonged and repeated use, even at a low concentration, is sufficient to exert selection pressure on the bacterial community to maintain their “resistome” and their spillover to human beings (57). Asian countries share the majority of global fish production, with China alone contributing to more than 60%. Little is known about the amount of AMU in aquaculture, particularly in LMICs due to a lack of strict monitoring, however, it varies depending upon geographical areas, climate, disease prevalence, and other socioeconomic factors. A single hatchery in Bangladesh experiences about 80 kg of antibiotics per cycle, a recent study reported (58). In Vietnam, about 72% of aquaculture farms used ~3.3 g of antimicrobial per kg of fish/shrimp product in the form of pre-medicated feed (59). Metal-based antifouling compounds (biocides) which are in use in aquaculture to prevent or treat bacterial or parasitic diseases may confer co- or cross- resistance to many antimicrobials (60). Fish are the most affordable source of protein in LMICs, with low cholesterol and high fatty acids, as they are cheaper price than beef, chevon, or poultry meat. An increased protein demand to boost immunity during the COVID period, especially in infants, children, and the elderly, can be met by fish due to their easy digestibility. This increased demand will boost the commercial aquaculture farms, possibly encouraging the use of more antibiotics.

## Household Use of Antimicrobials

Various scientific and medical bodies relied upon alcohol-based hand rub and handwashing with soap water as the most effective tools to combat the recent emergence of the COVID virus. Meanwhile, various commercial sanitizers, most of which contain medically important disinfectants, flooded the market with claims to effectively decimate the virus. The possible link between the use of disinfectants and the development of AMR received wide attention when the Food and Drug Administration (FDA) banned the use of triclosan in antibacterial soap (61). Sodium hypochlorite, commonly used in household cleaning or sewage decontamination and chlorination of water, has been on the news recently for its controversial use in purportedly eliminating coronavirus from the human body. Chlorination-induced oxidative injury was reported to increase resistance in *Pseudomonas aeruginosa* against ceftazidime, chloramphenicol, and ampicillin by 1.4–5.6-fold through overexpression of the MexEF-OprN efflux pump (62). Benzalkonium chloride (BAC) is a quaternary ammonium used as a preservative in eye, ear, or nasal drops. Lately, BAC was found to induce resistance even against last-resort antibiotics like polymyxin (63). There are reports of other quaternary ammonium compounds at sub-inhibitory concentrations affecting the susceptibility of *E. coli* strains to diverse antimicrobials, such as phenicol compounds, β lactams, and quinolones (64). There is conflicting evidence on the role of alcohol-based hand sanitizers (ABHS) in the spread and development of antibiotic-resistant bacteria. The repeated and prolonged use of ABHS may lead to the rise of alcohol-tolerant *Enterococcus faecium* in hospital environments due to mutations in carbohydrate uptake and regulation genes (65). Use of alcohol hand rubs was also reported to facilitate the growth of multidrug-resistant strains of *Acinetobacter baumannii* (65).

## Categories of Antimicrobial Resistant Bacteria Prevalent in Agri-Food Chain and Companion Animals in LMICs

### Livestock-Associated Methicillin-Resistant *Staphylococcus aureus* (LA-MRSA)

Livestock-associated methicillin-resistant *Staphylococcus aureus* (LA-MRSA) is an emerging pathogen that has been detected all over the world and, like many other bugs, its presence and spread are supported by the AMU and hygiene in addition to some other under-reported/poorly investigated factors like farm size, farming system, use of disinfectants, and in-feed zinc (66). Initially reported in animals, LA-MRSA is being increasingly reported in human beings; prolonged and frequent exposure of the farm-workers increased the risk of zoonotic transmission. Reservoir animals as asymptomatic carrier cannot be detected unless screened for MRSA, which is not under a routine surveillance program in any of the LMICs. Thus, we need to rely on the sporadic reports available from these countries. A number of studies from China demonstrated LA-MRSA infection as an occupational hazard for pig-farm workers (67), although such a possibility is very low (68). However, depending upon the prevailing farming style, nasal colonization of LA-MRSA in pig handlers may vary country wise (i.e., 5.5% in Malaysia, 15% in China, and 19.2% in Taiwan) (69). Unlike European and North American countries, which witnessed wide-spread detection of ST-398 clonal type of LA-MRSA, ST-9 predominates in the Asian continent. The majority of the LA-MRSA strains belong to SCCmec type IV and V and were frequently co-resistant to tetracycline and lincosamide, mediated by *tet* and *erm* genes, respectively (70), and glycopeptide-resistance remained a rare finding (71). MRSA needs special attention, as previously a SARS-CoV epidemic caused a significant rise in MRSA infection, especially in patients who required ventilation support (72). The recent episode of COVID19 may also produce a similar situation, as a large number of patients, especially with comorbid conditions, required ventilation support in the ICUs.

### Cephalosporin Resistant Bacteria

Due to mutated or modified penicillin binding protein (PBP2a), MRSA are resistant to all β-lactam drugs, including cephalosporins. The Gram-negative bacteria (GNB), which are cephalosporin-resistant mainly through the production of β-lactamase or cephalosporinase- like AmpC type β-lactamase (ACBL) and extended-spectrum β-lactamase (ESBL), have become a cause of concern for their global spread, high infectivity, and associated mortality. Food and companion animals being the reservoir of ESBL or ACBL producers can put people in contact with them or consumers at significant risk. Due to abysmal public health infrastructure and poor hygiene, LMICs are overburdened with neonatal sepsis and healthcare-associated infections (73), with a heavy load of such drug-resistant pathogens in hospitals and communities (74). About 22% of healthy humans from Southeast Asia were found to harbor ESBL-producing bacteria in their gut-which is much higher than the global average (14%) (75). Increased contact with food animals is often assumed as an important underlying determinant for this higher ESBL colonization in Asian and African population (76). A number of studies from LMICs implicated food-animals as important reservoirs of ESBLs, however, their role in human infection cannot be confirmed. In contrast, chickens were reported to acquire ESBL resistance from water contaminated with human sewage through integrated farming, which is a common practice in many parts of Southeast Asia (77). In cows, ESBL-producers with multiple CTX-M variants were reported from mostly fecal sources and mastitic milk with a preponderance of CTXM-1 and CTXM-15 variants. Among other common types of the ESBLs in bovines from various LMICs were SHV-180, OXA-10, SHV-5 (Turkey), SHV-12 (Turkey and Egypt), SHV-180 (India), OXA-30 (Egypt), and TEM (India) (78). A number of studies conducted by our group revealed the presence of some ESBL variants in bovine mastitis wherein we could not trace any known ESBL genes by PCR screening – existence of a novel ESBL mechanism in this part cannot be ruled out (79, 80). Cattle manure is widely used as biological fertilizer, which increases the chance of environmental dissemination of ESBLs from a fecal source. Separate studies conducted by our group revealed the presence of ESBLs in broilers or birds kept exclusively for meat (81, 82) but not in backyard poultry reared by a large section of resource-poor farmers in India (83). Likewise, ESBLs and ACBLs were frequently detected in poultry from the developing countries of Asian (84, 85), African (86), and European (87) regions. Importantly, pigs, which form a favorite and affordable dish in many protein-hungry countries, were reported to carry ESBL determinants (88–90). The ESBL detection rate was much higher in chicken meat (~93%) than meats from other animals, such as pork, beef (~35%), and fish/shrimps (~29%), in a study conducted on multiple species in Vietnam; possibly contamination with the caecal flora and poor disinfection measures led to this higher detection rate (91).

### Carbapenem Resistant Bacteria

Carbapenems, due to their exceptional ability to withstand drug-resistance mechanisms like ESBLs, have remained an automatic choice of clinicians for treating refractory infections caused by ESBL-producing GNB. The emergence of carbapenem-resistance, which is often transmitted via mobile genetic elements, has far-reaching and rippling consequences, particularly in countries like India (92), China, and other Southeast Asian countries (50, 93) with heavy loads of ESBL infection in the healthcare setting. Carbapenem resistant *Enterobacteriaceae* (CRE) has serious repercussions in LMICs like many other bugs and infections, as it makes the poor vulnerable due to their lack of access to healthy environments, hygiene, and safety measures. Poor socio-economics put people at higher risk of contracting carbapenem-resistant pathogens (93) and, like many other bugs, this high priority pathogen was found to emanate amid poverty, violence, discrimination, and weak governance – key characteristics of LMICs (94). Fortunately, carbapenem is never chosen by veterinarians for treating food animals, possibly because of the cost and regulations putting a bar on its use. This is probably why carbapenem resistance has been rarely reported among food animals, particularly from LMICs. However, animals can also be exposed to such drugs or bugs in the environment through contaminated and untreated wastewater discharges from hospitals; a lack of proper AMR surveillance in LMICs may be an important reason for the underreporting of CRE in food animals. In India, in two separate instances, bovine mastitis was reported to carry NDM-5 producing *E. coli* (95, 96). Further, two different studies – one in Algeria (97) and another in the Jiangsu province of China (98) - reported NDM-5 producing *E. coli* and NDM-5 producing *K. pneumoniae*. Again from China, recently, another study reported an NDM-5-producing *Escherichia coli* strain in poultry (99), indicating an insidious spread of CRE across food animals; however, proper surveillance is required to unveil it. Arguably, NDM-5 seems to predominate in animals - not only in food animals as discussed above, but also in pets as reported from three continents: Africa (100), Asia (50), and North America (101). Recent analysis of the COVID scenario indicated how resistant pathogens, such as carbepenem-resistant and cephalosporin-resistant *Enterobacteriaceae*, may complicate the SARS-CoV2 pneumonia (102). Overenthusiastic antibiotic therapy in managing COVID patients and chemoprophylaxis may promote these pathogens further.

### Colistin Resistant Bacteria

Colistin, a near abandoned drug due to its nephrotoxicity, has once again begun gaining attention for its usefulness in treating extremely drug-resistant pathogens like carbapenem-resistant *Enterobacteriaceae* (103). Plasmid mediated colistin-resistance (*mcr*) was first reported from China, linked with the use of colistin in pig and poultry farming (29). Surprisingly, colistin was not a banned drug in veterinary practices even in many high-income countries (HICs) until the emergence of *mcr*. Till date, *mcr* and its different allelic variants have been reported from pigs, poultry (104), cattle (105), and companion animals (106), but not in human beings. Colistin-resistance was known to be mediated by chromosomal modification in the two component regulatory system or deletion of *mgr* (107); however, the plasmid-mediated colistin-resistance has struck the medical community for the possibility of its rapid spread. Since its initial detection in 2016 from China, plasmid-mediated colistin-resistance has been reported from different LMICs, such as Egypt (108), India (109), Vietnam (110), Brazil (111), and Argentina (106). Importantly, as the whole world is searching for an effective weapon against coronavirus, an *in silico* model predicted the ability of colistin to interfere with the function of novel coronavirus by interacting with the viral aminoacid residue pockets (Thr24-Asn28 and Asn119) through hydrogen bonds (112).

## Role of Animal/Poultry Farms as Reservoir of AMR Bacteria in LMICs

The human population is expected to grow by 50% by the year 2050 (113), with consequential increases in the demand for food. In LMICs, consumer preference shifted toward animal protein from vegetables, consistent with enhanced income, urbanization, and demographic and lifestyle changes (114). While the global meat consumption is expected to rise by 76% between 2,000 and 2,050, the rise in LMICs is more than 200% due to an increased population with enhanced per capita consumption (115). Only in South-East Asian (SEA) countries is the demand for poultry projected to increase by 725% between 2,000 and 2,030 (116).

### Industrial Food Animal Production (IFAP)

The Industrial Food Animal Production (IFAP) has witnessed a massive growth to meet the rising demand for animal protein. Because of intensive rearing, higher stocking density, zero-grazing, overdependence on MIA and non-MIA for therapy, prophylaxis and growth promotion, and poor waste management (117), IFAP is not without hazards, such as offensive odors, increasing risk of zoonoses, including AMR, and non-communicable disorders such as stress, hypertension, and cognitive impairment among animal handlers and people in the surrounding community (118). The only benefit of IFAP, as argued by the Brazilian Government, is less environmental degradation, such as reduced deforestation (119).

In LMICs, due to the rising demand and expansion of multi-national food production companies, the IFAP is slowing replacing the small-scale backyard/household rearing system (120). IFAP is preferred particularly in urban areas of countries like Ethiopia, Uganda, and Vietnam that experience a shortage of land and water (121). A lack of stringent regulations and monitoring increased the non-therapeutic use of antimicrobials in farms (mostly pig and poultry) in several LMICs, such as China (122), Vietnam (123), Ethiopia (124), Uganda (125), Kenya (126), Mexico (127), and Myanmar (128).

The persistence of antimicrobia- resistant bacteria in IFAP settings is associated with several factors, such as AMU, co-resistance, cross-resistance with heavy metals, bacterial fitness, mixing of new and old animals, vectors or reservoirs of bacterial infection, vertical and pseudo-vertical transmission, and cleaning and disinfection (129). Even animal transport vehicles and flies originated from IFAP play a major role in the transmission of AMR into the community (130).

### Backyard Farming

Unlike the developed world, LMICs are largely dependent upon small-scale backyard farming and as a result are more environment-friendly; backyard farming is regarded as sustainable even after meeting the rising demand for animal protein. As the animals are kept in small flocks or herds and maintained in a free-range system with occasional supplementations of raw vegetables with minimum manpower, backyard farming poses a relatively low risk for zoonotic transmission (131). However, small-scale backyard farming (chicken and pigs) is converting rapidly into “medium- to large-scale” backyard faming by making agreements with different food companies (“contract farming”).

In general, backyard farming is operated with minimal antimicrobial intervention, replaced instead by indigenous technical knowledge (ITK) or lower generation cheaper antibiotics (132). Sporadic studies in different LMICs (Tanzania, Ecuador, Vietnam, Ghana, Bangladesh, and Cambodia) revealed the usage of antimicrobials like oxytetracycline, penicillin, erythromycin, enrofloxacin, and trimethoprim-sulfadozine by “medium- to large-scale” backyard pig and chicken farmers (77, 133), based on personal experience or communication without veterinary oversight. The lack of costly higher generation cephalosporin usage in backyard household poultry was reflected in the absence of extended spectrum beta-lactamase determinants in *Salmonella* and *E. coli* isolated from backyard layers in India (83, 134).

The use of AMUs by farmers in backyard farms is influenced by their capacity to detect the diseased animals, the farmer's expertise and attitude toward the disease-associated risk, and the cost-benefit analysis of treatment (135). The cheaper variety of the antimicrobials is always preferred, although sometimes it is unsafe due to compromising with the quality, especially in LMICs. The overall prevalence of low-quality medicine was estimated to be 13.6% in LMICs, and further, 12.4% of the antibiotics were substandard or falsified (136). The annual market for unregistered and poor quality veterinary drugs in Africa is estimated to be equal to the registered drug market (400 million US dollars) (137). The bacterial population exposed to the poor quality veterinary medicine is not wiped out completely due to sub-therapeutic dosages and the ineffective release of drugs, and the left over bacteria may remain as a resistant population with subsequent transmission (138).

## Mitigation Strategy

### Substitution of AMU

AMU is the single most important driver for AMR; therefore, attempts are being made to slowly reduce or phase out antimicrobials in veterinary medicine. However, substitution of antimicrobials has short-term economic implications resulting from substantial loss of production and higher morbidity or mortality. In any case, if such measures compromise food security, that may have devastating impacts in poor, highly populous, and resource-deprived countries. An early warning system based on a local epidemiological database and regular health check-ups of the animals may allow us to detect the disease early and thereby prevent its spread to other animals in the herd or adjoining areas. Thus, widespread chemoprophylaxis that becomes indispensable in any outbreak may be avoided. This needs to be revisited in the face of the COVID outbreak, with several pets and wild animals having tested positive for SARS-CoV-2, like cats, dogs, tiger, lions, minks, ferrets, hamster, bats, and macaques. In addition, preventive non-antimicrobial strategies which include- timely vaccination, appropriate biosecurity measures, proper nutrition and housing may reduce the demand for preventive antimicrobial therapy. The adoption of herd-specific control measures to minimize the occurrence of diseases like mastitis may be helpful to promote the prudent use of antimicrobials (139).

Various alternative ways, like reducing meat consumption, capping the amount of antimicrobials per year per kilogram of animal product, and making antibiotics expensive by taxation, were proposed to cut down AMU in food animals (140). Some sort of economic shield in the form of insurance packages or incentives to safeguard the loss arising out of any infectious diseases in the farm may psychologically motivate farmers to use antibiotics more judiciously (135). Making the meat from antibiotic-treated animals more expensive and labeling such meat packages with its source (from antibiotic-treated animals) is another way to discourage consumers and to indirectly reduce AMU (141).

The alternative anti-infective strategies, such as nano-material based anti-infective particles, enzymes, antimicrobial peptides, quorum sensing quenchers, efflux pump inhibitors, clay, predatory bacteria, teat sealants, and antimicrobial photodynamic therapy, are in the pipeline to be evaluated at a field level. The supplementation of essential oils and spices as an alternative to antimicrobials was reported to have beneficial health effects in poultry (142, 143). However, the performance of essential oils still needs to be clinically tested in various conditions and they may not be equally effective against the Gram-negative pathogens because of inherent tolerance (144); the absence of maximum residue levels (MRL) data regarding those essential oils in food animals has to be checked.

### Raising Awareness Among Farmers

FAO referred to farmers as “important frontline defenders” for the vital role they can play in stemming the spread of AMR by adopting good hygienic farm operations. Increasing awareness among farmers by imparting basic knowledge may help reduce the unnecessary and indiscriminate AMU in food animals (104); however, it can only be successful if adequate financial support and insurance packages are given to recuperate any loss in livestock farming (145). In most of the LMICs, small and marginal farmers often suffer huge economic losses due to disease outbreaks for meager investments on biosecurity and farm hygiene; these psychologically disadvantaged farmers can then be easily misled about the purported efficacy of antimicrobials for growth promotion and disease prevention by the unscrupulous push-sell of drug-marketing agencies. Inadequate veterinary healthcare facilities and limited drug regulations increase the magnitude of the problem in LMICs and, without reshaping these, efforts to bring sustainable change in farmers' behavior, knowledge, attitude, and practices may be futile (145). Changing farmers' behaviors or increasing their awareness for appropriate AMU in food animals requires multiple supportive measures, like incentives to the farmers raising livestock without antibiotics, subsidized insurance to make up for losses, the implementation of strict drug regulation, and the establishment of a strong network of veterinary healthcare facilities accessible to rural farmers in LMICs. The mobile veterinary clinic was introduced by the Government of India to reach out to the country's remote corners.

### Implementation of Government Legislation

FAO underscored strong government legislation as the most important component in addressing the overuse, misuse, or abuse of antimicrobials that accounted for the rise in AMR. Such legislations are essential for defining the responsibilities and duties of all the stakeholders, and for the sustainability of the policies and technical objectives aimed at reducing AMR.

Drug regulatory agencies of European countries and the FDA implemented strong regulations by banning the prophylactic and growth-promoting use of antimicrobials in food animals and capping the limit of CIA/HIA in the veterinary sector. Many countries (Japan, the USA, Colombia, Denmark, the Netherlands, and Sweden) fixed national targets to reduce AMU in livestock. Nevertheless, any kind of regulatory endeavor is still at a primary stage in many of the LMICs (146); only a few could successfully implement such regulations or advisories given the prevailing socio-economic scenario, public administrative constraints, and absence of political commitment/goodwill in these countries. Therefore, emphasis on education, awareness, and training of all stakeholders, particularly the end users, might be more effective in LMICs (147).

### Surveillance of AMU and AMR

Surveillance on AMR pathogens and AMU is undeniably the key driving force for controlling AMR, with WHO suggesting this point in the global action plan on antimicrobial resistance (GAP), which still remains the authentic source of information to fight against AMR. Many of the HICs (Norway, Japan, Denmark, Canada, the USA, Finland, the Netherlands, and France) have articulated national surveillance programs (NORM-VET, JVARM, DANMAP, CIPARS, NARMS, FINRES-VET, NethMap-MARAN, ONERBA- RESAPATH, and SWEDRES-SVARM) and the policies on AMU in animals are tailored based on the data generated from their networks. Such is not the case in LMICs, as most of them have no surveillance in place to monitor the antimicrobial consumption in animals. The government of India adopted the NAP on AMR and strongly recommended the need for a strong regulatory framework for restricting the AMU in animals. A Pan-India Network – ICAR-Indian Network for Fisheries and Animal Antibiotic Resistance (INFAAR) was initiated by the Indian Council of Agricultural Research in collaboration with FAO to cater to the objectives laid down in India's NAP on AMR. A lack of robust infrastructural support crippled by financial constraints remains the most pressing challenge for the establishment and proper functioning of a robust surveillance system on AMR/AMU in LMICs. On the contrary, Ashley et al. (148) proposed to tap the large amount of data generated by academic institutes and private laboratories to indirectly and passively monitor the problem in these areas for the time being.

### Drug-Repurposing Strategy

The COVID19 panorama has been an eye-opener for scientists worldwide; when there are no or limited therapeutic choices, with a new drug or effective vaccine still a long way off, the only option left is to experiment with the existing modalities. The whole world is frantically searching for a solution through drugs such as hydroxychloroquine, azithromycin through ivermectin (149), famotidine (150), flavipiravir (151), and remdesivir (152). This is the same situation with novel antimicrobials as the plausibility of a new drug to hit the market in the near future is remote. Even if it comes to market, how long it will be effective for is not clear. The search for new antimicrobials is impeded by the huge investment requirement, time lag, and reluctance of pharma-leaders recently shifting their focus toward cheaper strategies like the repurposing of drugs which involves less time and investment. No such study has been conducted on repurposing of drugs in the veterinary sector. However, veterinary drugs were tried for repurposing; fenbendazole was found to be effective against non-small cell lung cancer cells (153) by microtubule destabilization and inhibition of glucose uptake. Likewise, isoxazoline was found to be promising in human vector-borne diseases. Many anthelmintic compounds of the salicylanilide family-niclosamide, oxyclozanide, rafoxanide, and closantel - demonstrated antibacterial properties against a wide range of pathogens -methicillin-, vancomycin-, linezolid-, or daptomycin-resistant *Staphylococcus aureus, Clostridium difficile, Klebsiella pneumoniae, Pseudomonas aeruginosa, Acinetobacter baumannii*, and *Helicobactor*. Avermectins were tested successfully against *Mycobacterium* and MRSA. Likewise, an antifungal property was reported for mebendazole. Trials with a few NSAIDs (Celecoxib, aspirin, ibuprofen, and tacrolimus) against a few bacterial and fungal pathogens turned out hopeful (154). A number of studies were conducted using anticancer drugs to repurpose as antibacterial, 5-fluorouracil and gallium nitrate were found to be effective against MDR *A. baumanni* and *Pseudomonas*. Tamoxifen, floxuridine, and streptozotocin exhibited appreciable antibacterial activity against *Staphylococcus* isolates.

However, the dose of the repurposed drugs is comparatively higher when used as an antimicrobial, so the pharmacokinetic profiles changes abruptly which necessitates a clinical revaluation and toxicity testing. LMICs, where the burden of infection is quite high, can provide ample scope for such trial and testing with funding support from international agencies.

### Pivotal Mitigation Strategy to Be Focused in Post COVID Period

The pos- COVID scenario might be associated with a rise in AMR in human and animals due to more stress on antibiotic-dependent healthcare systems to combat secondary bacterial infections. Interruptions of antibiotic stewardship programs in hospitals and communities, prescription with antibiotics for COVID patients misdiagnosed with bacterial bronchitis, over-prescription in telehealth consultations, and easy accessibility of medicines from online pharmacy are just a few key factors associated with AMR identified during the pandemic (155). Enhancement of the immune system with an increased animal-based protein diet was promoted by governments and non-governmental organizations throughout the world during the pandemic. It will further increase the demand for animal protein, which may enhance the growth promotional use of antimicrobials in IFAPs.

Antimicrobial resistance is mostly dependent on the use of antibiotics in humans, agri-food chains, and companion animals, and the use of antibiotics is largely regulated by human instinct (156). The knowledge, attitude, and practice of the AMU by all types of prescribers and farmers varies a lot between developed countries and LMICs, which will be further modified with added complexities during a post-pandemic period. The qualitative and quantitative survey should be established at a national level in LMICs to explore the behavioral basis of AMU during the post-COVID period. A national level monitoring system should be established for the quantification of AMU categorically in different species of animals, birds, fish, and agri-products to detect the risk factors for the emergence of any change in resistance pattern post the pandemic. The quantification of AMU will develop a benchmarking system with an immediate identification of the top-level user, although reduction of AMU is not always directly correlated with reduction of AMR, as it is a multi-factorial issue (as described earlier). More farm-level molecular epidemiological studies in livestock, poultry, and aquaculture to identify the reservoir of resistant bacteria, categorize the resistance determinants, establish the correlation between resistance determinants and true resistance against MIA, explore the environmental resistome, and explore the wildlife as carrier of resistant bacteria are in dire need. A holistic one health approach based intervention strategy incorporating all the local stakeholders of each LMIC is required to address the complex issue after identification of the major driver which may vary between the member countries and during the pre- and post-pandemic periods. The one health approach to address the AMR issue might be an easier process during the post-COVID period as several international collaborative groups were already created during the pandemic and, moreover, political will and subsequently more research and development investments by governments to address health-related issues is expected to save the human population.

## Author Contributions

SB and IS planned and wrote the manuscript. All authors contributed to the article and approved the submitted version.

## Conflict of Interest

The authors declare that the research was conducted in the absence of any commercial or financial relationships that could be construed as a potential conflict of interest.
